# Exploring Characteristics of Caregivers Who are Financially Dependent on Their Care Receivers

**DOI:** 10.1093/geroni/igaf122.2652

**Published:** 2025-12-31

**Authors:** Eleanor Batista-Malat, Kathleen Wilber, Laura Mosqueda, Bonnie Olsen

**Affiliations:** University of Southern California, Los Angeles, California, United States; University of Southern California, Los Angeles, California, United States; University of Southern California, Pasadena, California, United States; University of Southern California, Los Angeles, California, United States

## Abstract

Financial and emotional dependence of a caregiver or other trusted person on an older adult is a risk factor for elder abuse. However, there is little investigation of the personal characteristics associated with caregivers who are financially dependent on care receivers. In this secondary analysis of a convenience sample of caregivers of people living with dementia (N = 76), we examined characteristics that may be associated with financial dependence, drawing on findings on dependent caregivers and high-risk abusers. A majority of the caregivers were women (67%), Latino (50%), and lived with the care receiver (58%); a plurality was caring for a parent (47%). Bivariate test show that financially dependent caregivers (24%) had higher rates of conflict with the care receiver before dementia (28% vs 9%, p=.036), use of alcohol to cope (33% vs 5%, p=.001), and payment for caregiving (33% vs 9%, p=.009). There were no differences in the amount of caregiving, depression and anxiety symptoms, positive/neutral coping methods or smoking to cope, burden, or demographic characteristics. Financial dependence was not associated with physical or psychological mistreatment but was associated with neglect (39% vs 14%, p=.019). Our findings suggest that financially dependent caregivers provide support to people living with dementia while facing their own challenges and engaging in neglect. Policies designed to support these caregivers in building skills without removing them from their role could bolster older adults’ networks of family caregivers and reduce caregiver neglect.

